# Severe anemia, severe leukopenia, and severe thrombocytopenia of amphotericin B deoxycholate-based induction therapy in patients with HIV-associated talaromycosis: a subgroup analysis of a prospective multicenter cohort study

**DOI:** 10.1186/s12879-023-08394-7

**Published:** 2023-10-20

**Authors:** Yihong Zhou, Tao Lu, Yan Li, Yuanyuan Qin, Yanqiu Lu, Qun Tian, Ke Lan, Guoqiang Zhou, Yingmei Qin, Vijay Harypursat, Shunmei Li, Shide Lin, Yaokai Chen

**Affiliations:** 1 Department of Infectious Diseases, Affiliated Hospital of Zunyi Medical University, Guizhou, China; 2 Division of Infectious Diseases, Chongqing Public Health Medical Center, Chongqing, China; 3 School of Public Health, Zunyi Medical University, Guizhou, China; 4 Division of Infectious Diseases, the Third People’s Hospital of Guilin, Guangxi, China; 5 Division of Infectious Disease, Longtan Hospital of Guangxi Zhuang Autonomous Region, Guangxi, China; 6 Division of Infectious Diseases, the First Hospital of Changsha, Hunan, China; 7 Division of Infectious Diseases, the Fourth People’s Hospital of Nanning, Guangxi, China; 8 Department of Cardiology, Affiliated Hospital of Zunyi Medical University, Guangxi, China; 9 College of Laboratory Medicine, Zunyi Medical University, Zunyi, China

**Keywords:** HIV, Talaromycosis, *Talaromyces marneffei*, Anemia, Leukopenia, Thrombocytopenia, Risk factors

## Abstract

**Background:**

This study’s objective was to investigate the predictors for severe anemia, severe leukopenia, and severe thrombocytopenia when amphotericin B deoxycholate-based induction therapy is used in HIV-infected patients with talaromycosis.

**Methods:**

A total of 170 HIV-infected patients with talaromycosis were enrolled from January 1st, 2019, to September 30th, 2020.

**Results:**

Approximately 42.9%, 20.6%, and 10.6% of the enrolled patients developed severe anemia, severe leukopenia, and severe thrombocytopenia, respectively. Baseline hemoglobin level < 100 g/L (OR = 5.846, 95% CI: 2.765 ~ 12.363), serum creatinine level > 73.4 µmol/L (OR = 2.573, 95% CI: 1.157 ~ 5.723), AST/ALT ratio > 1.6 (OR = 2.479, 95% CI: 1.167 ~ 5.266), sodium level ≤ 136 mmol/liter (OR = 4.342, 95% CI: 1.747 ~ 10.789), and a dose of amphotericin B deoxycholate > 0.58 mg/kg/d (OR = 2.504, 95% CI:1.066 ~ 5.882) were observed to be independent risk factors associated with the development of severe anemia. Co-infection with tuberculosis (OR = 3.307, 95% CI: 1.050 ~ 10.420), and platelet level (per 10 × 109 /L) (OR = 0.952, 95% CI: 0.911 ~ 0.996) were shown to be independent risk factors associated with the development of severe leukopenia. Platelet level < 100 × 10^9^ /L (OR = 2.935, 95% CI: 1.075 ~ 8.016) was identified as the independent risk factor associated with the development of severe thrombocytopenia. There was no difference in progression to severe anemia, severe leukopenia, and severe thrombocytopenia between the patients with or without fungal clearance at 2 weeks. 10 mg on the first day of amphotericin B deoxycholate was calculated to be independent risk factors associated with the development of severe anemia (OR = 2.621, 95% CI: 1.107 ~ 6.206). The group receiving a starting amphotericin B dose (10 mg, 20 mg, daily) exhibited the highest fungal clearance rate at 96.3%, which was significantly better than the group receiving a starting amphotericin B dose (5 mg, 10 mg, 20 mg, daily) (60.9%) and the group receiving a starting amphotericin B dose (5 mg, 15 mg, and 25 mg, daily) (62.9%).

**Conclusion:**

The preceding findings reveal risk factors for severe anemia, severe leukopenia, and severe thrombocytopenia. After treatment with Amphotericin B, these severe adverse events are likely unrelated to fungal clearance at 2 weeks. Starting amphotericin B deoxycholate at a dose of 10 mg on the first day may increase the risk of severe anemia but can lead to earlier fungal clearance.

**Trial registration:**

ChiCTR1900021195. Registered 1 February 2019.

## Background

Talaromyces marneffei, a prevalent dimorphic fungus among people living with HIV/AIDS in China (prevalence ranging from 3.3 to 15%) [1, 2], has a mortality rate of up to 30% [3], particularly in older patients [4], despite appropriate anti-fungal treatment. Anemia is commonly present in HIV-infected patients with talaromycosis, and its prevalence is between 80 and 95.6% [5, 6]. This could lead to negative outcomes, such as prolonged hospitalization, a requirement for close monitoring, and eventually the requirement for blood or platelet transfusion [7]. Thrombocytopenia and anemia are both independent risk factors for poor prognosis in HIV-infected patients with comorbid talaromycosis [8–10]. Moreover, platelets have been shown to possess antimicrobial activity against bacteria, viruses, and fungi [11, 12]. Leukopenia occurs in approximately 40% of HIV-infected patients with talaromycosis [5], and can weaken the host immune system, increasing the susceptibility of patients to opportunistic infections [8]. This susceptibility may ultimately impact the progression of HIV-associated talaromycosis. Currently, it is unclear which risk factors predict the development of severe anemia, severe leukopenia, and severe thrombocytopenia in HIV-infected patients with talaromycosis, particularly in those undergoing treatment with amphotericin B deoxycholate.

Current guidelines recommend amphotericin B deoxycholate as the preferred induction therapy for talaromycosis. An open-label, non-inferiority trial conducted in Vietnam found that amphotericin B deoxycholate was superior to itraconazole as initial treatment [3]; however, the toxic effects of amphotericin B deoxycholate, such as anemia, leukopenia, and thrombocytopenia, cannot be ignored [13, 14]. Up to 40% of HIV-infected patients with talaromycosis, whether with anemia or not at baseline, develop severe anemia after they have initiated amphotericin B deoxycholate [3]. The incidence of patients who started amphotericin B deoxycholate and subsequently developed severe leukopenia and severe thrombocytopenia is approximately 10% and 20%, respectively [3]. We recently completed a prospective multicenter cohort study evaluate efficacy and safety of voriconazole versus amphotericin B deoxycholate induction treatment for HIV-Associated talaromycosis [15]. Up to 45% of HIV-infected patients with talaromycosis in the amphotericin B deoxycholate group had a hemoglobin level below 74 g/L, which is higher than the voriconazole group. We are uncertain whether the decrease in hemoglobin, leukopenia, and thrombocytopenia observed in HIV-infected patients with talaromycosis treated with amphotericin B deoxycholate is caused by the fungi or amphotericin B deoxycholate. The risk factors which predict severe anemia, severe leukopenia, and severe thrombocytopenia in HIV-infected patients comorbid with talaromycosis being treated with amphotericin B deoxycholate are unknown. We, therefore, use data from a multi-center prospective observational study to assess risk factors for the occurrence of severe anemia, leukopenia, and thrombocytopenia in HIV-infected patients being treated with amphotericin B deoxycholate for talaromycosis.

## Methods

### Study design and setting

This was a prospective, multi-center, observational study of HIV-infected patients with talaromycosis who were admitted to hospitals between January 1st, 2019, and September 30th, 2020. The study enrolled patients from 11 hospitals located in 9 cities, namely: Chongqing Public Health Medical Center, Guangzhou Eighth People’s Hospital, Guangxi Longtan Hospital of Guangxi Zhuang Autonomous Region, Liuzhou General Hospital, the Third People’s Hospital of Guilin, the First Hospital of Changsha, the Fourth People’s Hospital of Nanning, Kunming Third People’s Hospital, Guiyang Public Health Clinical Center, Beijing Youan Hospital of Capital Medical University, and Yunnan Provincial Infectious Disease Hospital. Eligible patients were adults aged 18 years or older with confirmed HIV infection and confirmed talaromycosis by either microscopy or culture. Exclusion criteria included patients with hematologic diseases causing anemia (including aplastic anemia, hemolytic anemia, bleeding, and so on), severe active infections caused by bacteria or other microbes, tumors, hemoglobin levels less than 80 g/L, leukocyte counts less than 1.0 × 10^9^/L, neutrophil counts less than 0.5 × 10^9^/L, platelet counts less than 30 × 10^9^/L, blood amylase levels greater than 2 times the upper limit of the reference level, serum creatinine levels greater than 1.5 times the upper limit of the reference level, aspartate aminotransferase (AST), alanine aminotransferase (ALT), or alkaline phosphatase levels greater than 5 times the upper limit of the reference level, total bilirubin levels greater than 2 times the upper limit of the reference level, and serum creatine phosphokinase (CK) levels greater than 2 times the upper limit of the reference level. Patients received treatment with amphotericin B deoxycholate for 14 days at a dose of 0.5 to 0.7 mg per kilogram per day, starting with smaller doses of amphotericin B deoxycholate (5 to 10 mg once a day) and gradually increasing the dose by 5 to 10 mg/day to the final daily dose. Written informed consent was obtained from all patients or their representatives. The independent ethics committees of each participating hospital approved the trial protocol.

### Assessments

We explored demographic factors, symptoms and signs, diagnosis of opportunistic infections, laboratory test results at admission, and types of combination antiretroviral therapy before diagnosis of talaromycosis. Each individual was invited to participate in 2-weeks of follow-up. At the follow-up visits at weeks 1, 2, hemoglobin, platelets, and leukocytes were evaluated.

### Outcomes and definitions

Patients were evaluated for hematological toxicity, including anemia, leukopenia, and thrombocytopenia at weeks 1 and 2 after the initiation of amphotericin B deoxycholate. The primary outcome was the occurrence of severe hematological toxicity due to any cause, defined as the appearance of at least one of three laboratory abnormalities during amphotericin B deoxycholate treatment [14]. Severe anemia was defined as hemoglobin level drop to 80 g/L. Severe leukopenia was defined as leukocyte count drop to 2 × 10^9^ /L or, if baseline count was under 2 × 10^9^ /L, a decrease of 25% in leukocyte count. Severe thrombocytopenia was defined as platelet count decrease to 50 × 10^9^ /L, or, if the baseline count was under 50 × 10^9^ /L, a decrease of 25% in platelet count.

Weight loss was defined as loss of more than 10% of body weight within 6 months. All patients were tested for tuberculosis, cytomegalovirus infection, syphilis, hepatitis B, and hepatitis C. These diagnoses were confirmed according to laboratory testing results. A large fraction of the diagnoses for pneumocystis pneumonia, oral candidiasis, and toxoplasma encephalopathy were presumptive, due to easier establishment of a clinical diagnosis and the limited availability of definitive pathogenic testing for these specific diseases.

### Statistical analysis

Statistical Package for the Social Sciences software, Version 25.0 (IBM-SPSS Statistics, Armonk, New York, USA) was used to analyze all study data. Standard descriptive statistics analyzed the clinical characteristics, diagnosis, and laboratory variables of patients. Continuous variables and categorical variables were compared using the Mann-Whitney U test and Chi-squared tests, respectively. Continuous variables with a *p*-value of ≤ 0.1, such as BMI, serum creatinine, AST/ALT, sodium, and potassium in the analysis of severe anemia and platelet level in the analysis of severe leukopenia were converted to categorical variables by grouping values using cut-off points based on a receiver-operating characteristic curve (ROC). Continuous variables with a *p*-value of ≤ 0.1, such as hemoglobin levels, platelet counts, and potassium levels in the analysis of severe anemia were converted into categorical variables by clinically relevant values. In identifying independent factors associated with severe anemia, severe leukopenia, or severe thrombocytopenia, variables were initially analyzed using a bivariate model, and subsequently independent risk factors were identified by means of a logistic regression model using a forward, stepwise approach, which began with inclusion of all variables associated with severe anemia, severe leukopenia, or severe thrombocytopenia on bivariate analysis (*p* ≤ 0.1), and subsequently included only those variables with *p* ≤ 0.05 in the final model. We used the variance inflation factor (VIF) and tolerance value of each univariate predictor to make the Multicollinearity diagnosis. If the VIF was higher than 10.0 and the tolerance is lower than 0.1, the variable would not have been included in the multivariate analysis.

## Results

**Fig. 1:**
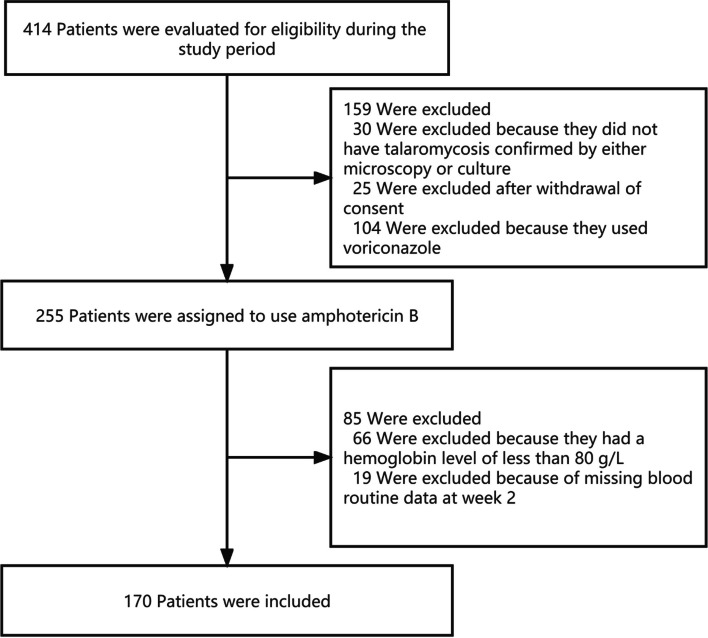
Study flowchart

During the study, 414 patients were evaluated for eligibility, and 170 of them were included (Fig. 1). 73 of 170 patients (42.9%) developed severe anemia, 35 of 170 patients (20.6%) developed severe leukopenia, and 18 of 170 patients (10.6%) developed severe thrombocytopenia after starting amphotericin B deoxycholate.

Table 1 shows baseline characteristics for patients. Compared with patients without severe anemia, more patients developed severe anemia had a lower BMI, weight loss, a lower hemoglobin level, a lower platelet level, a higher serum creatinine level, a higher AST/ALT ratio, a lower sodium level, and an induction therapeutic dose of amphotericin B deoxycholate of > 0.58 mg/kg/d (Tables 1 and 2). Compared with patients who did not develop severe thrombocytopenia, more patients developed severe thrombocytopenia had lower baseline platelet levels (Table 3).

**Table 1:** Clinical characteristics of the patients developing severe anemia or not at baseline

	Progression to severe anemia (*N* = 73)	No progression to severe anemia (*N* = 97)	*p*-value
**Socio-demographic**			
Female sex, n (%)	14 (19.2%)	10 (10.3%)	0.100
Age, median (IQR), years	43 (35–53)	42 (32–50)	0.173
Drink, n (%)	12 (16.4%)	17 (17.5%)	0.852
Smoke, n (%)	21 (28.8%)	27 (27.8%)	0.894
BMI, median (IQR), kg/m^2^	31 (29–35)	33 (30–36)	0.022
**Symptoms and signs**			
Fever, n (%)	59 (80.8%)	86 (88.7%)	0.153
Cough, n (%)	44 (60.3%)	52 (53.6%)	0.386
Sputum, n (%)	37 (50.7%)	38 (39.2%)	0.135
Hemoptysis, n (%)	2 (2.7%)	0 (0.0%)	0.183
Abdominal pain, n (%)	15 (20.5%)	15 (15.5%)	0.389
Diarrhea, n (%)	2 (2.7%)	8 (8.2%)	0.237
Headache, n (%)	3 (4.1%)	5 (5.2%)	1.000
Skin lesions, n (%)	21 (28.8%)	27 (27.8%)	0.894
Weight loss, n (%)	34 (46.6%)	24 (24.7%)	0.003
**Complications**			
Oral candidiasis, n (%)	29 (39.7%)	43 (44.3%)	0.548
Cytomegalovirus infection, n (%)	8 (11.0%)	9 (9.3%)	0.718
Tuberculosis, n (%)	8 (11.0%)	7 (7.2%)	0.394
Hepatitis B, n (%)	7 (9.6%)	4 (4.1%)	0.263
Pneumocystis pneumonia, n (%)	5 (6.8%)	8 (8.2%)	0.734
Syphilis, n (%)	3 (4.1%)	1 (1.0%)	0.424
Hepatitis C, n (%)	0	1 (1.0%)	1.000
Toxoplasma encephalopathy, n (%)	1 (1.3%)	0	0.429
**Laboratory results**			
Leukocyte, median (IQR), ×10^9^ /L	4.04 (2.78–5.02)	3.94 (2.67–5.63)	0.611
Hemoglobin, median (IQR), g/L	89 (84–102)	107 (97–116)	< 0.001
Platelet, median (IQR), ×10^9^ /L	111 (61–211)	151 (85–218)	0.041
CD4 + T-cell counts, median (IQR), cells/µL	15 (7–39)	12 (6–35)	0.510
Serum creatinine, median (IQR), µmol/L	68.90 (55.85–84.15)	61.00 (52.45-73.00)	0.018
Total bilirubin level, median (IQR), 10.5 mg/dl	10.57 (6.94–20.69)	8.9 (6.9–12.4)	0.144
AST/ALT ratio, median (IQR)	2.03 (1.47–4.03)	1.53 (1.16–2.34)	< 0.001
Sodium, median (IQR), mmol/liter	133.1 0(128.50–135.00)	133.7 (131.45–138.40)	0.015
Potassium, median (IQR), mmol/liter	3.56 (3.27–3.88)	3.66 (3.39–4.08)	0.095
**HAART before diagnosis**			
Antiretroviral therapy, n (%)	5 (6.8%)	14 (14.4%)	0.120
3TC	5 (6.8%)	13 (13.4%)	0.169
TDF	4 (5.5%)	10 (10.3%)	0.257
EFV	5 (6.8%)	9 (9.3%)	0.568
NVP	0	2 (2.1%)	0.507
AZT	1 (1.4%)	1 (1.0%)	1.000
ABC	0 (0.0%)	1 (1.0%)	1.000
Lpv/r	0 (0.0%)	1 (1.0%)	1.000
DTG	0	1 (1.0%)	1.000
Elvitegravir	0	1 (1.0%)	1.000
Amphotericin B			
Induction therapy > 0.58 mg/kg/d	24 (32.9%)	19 (19.6%)	0.048

*BMI* Body Mass Index, *BUN *Blood urea nitrogen, *AST *Aspartate aminotransferase, *ALT *Alanine aminotransferase, *HAART *Highly active antiretroviral therapy, *3TC *Lamivudine, *TDF *Tenofovir, *EFV *Efavirenz, *NVP *Nevirapine, *AZT* Zidovudine, *ABC *Abacavir, *Lpv/r *Lopinavir and ritonavir, *DTG *Dolutegravir, *FTC *Emtricitabine

**Table 2:** Clinical characteristics of the patients developing severe leukopenia or not at baseline

	Progression to severe leukopenia (*N* = 35)	No progression to severe leukopenia (*N* = 135)	*p*-value
**Socio-demographic**			
Female sex, n (%)	6 (17.1%)	18 (13.3%)	0.761
Age level, median (IQR), year	44 (35–55)	32 (30–36)	0.402
Drink, n (%)	8 (22.9%)	21 (15.6%)	0.306
Smoke, n (%)	11 (31.4%)	37 (27.4%)	0.638
BMI level, median (IQR), kg/m^2^	32 (30–34)	32 (30–36)	0.509
**Symptoms and signs**			
Fever, n (%)	30 (85.7%)	115 (85.2%)	0.937
Cough, n (%)	19 (54.3%)	77 (57.0%)	0.770
Sputum, n (%)	15 (42.9%)	60 (44.4%)	0.866
Hemoptysis, n (%)	1 (2.9%)	1 (0.7%)	0.370
Abdominal pain, n (%)	5 (14.3%)	25 (18.5%)	0.558
Diarrhea, n (%)	2 (5.7%)	8 (5.9%)	1.000
Headache, n (%)	2 (5.7%)	6 (4.4%)	1.000
Skin lesions, n (%)	8 (22.9%)	40 (29.6%)	0.428
Weight loss, n (%)	12 (34.3%)	46 (34.1%)	0.981
**Complications**			
Oral candidiasis, n (%)	15 (42.9%)	57 (42.2%)	1.000
Cytomegalovirus infection, n (%)	3 (8.6%)	14 (10.4%)	1.000
Tuberculosis, n (%)	6 (17.1%)	9 (6.7%)	0.107
Hepatitis B, n (%)	4 (11.4%)	7 (5.2%)	0.341
Pneumocystis pneumonia, n (%)	3 (8.6%)	10 (7.4%)	1.000
Syphilis, n (%)	1 (2.9%)	3 (2.2%)	1.000
Hepatitis C, n (%)	0	1 (0.7%)	1.000
Toxoplasma encephalopathy, n (%)	0	1 (0.7%)	1.000
**Laboratory results**			
Leukocyte level, median (IQR),×10^9^ /L	4.0 (2.77–4.67)	3.94 (2.60–5.60)	0.828
Hemoglobin level, median (IQR), g/L	96 (86–111)	101 (88–112)	0.275
Platelet level, median (IQR),×10^9^ /L	110 (64–172)	143 (74–229)	0.068
CD4 + T-cell counts, median (IQR),cells/µL	9 (6–34)	15 (7–38)	0.233
Serum creatinine level, median (IQR), µmol/L	67.7 (52.8–77.6)	64 (53.9–77.0)	0.603
Total bilirubin level, median (IQR),mg/dl	8.9 (7.30–13.60)	9.38 (6.90-15.92)	0.673
AST/ALT ratio, median (IQR)	1.84 (1.29–2.88)	1.81 (1.27–2.65)	0.768
Sodium level, median (IQR), mmol/liter	133.7 (130.0-136.4)	133.4 (130.0-137.0)	0.804
Potassium level, median (IQR), mmol/liter	3.64 (3.3-4.0)	3.56 (3.27–3.86)	0.277
**HAART before diagnosis**			
Antiretroviral therapy, n (%)	3 (8.6%)	16 (11.9%)	0.804
3TC	2 (5.7%)	12 (8.9%)	0.737
TDF	1 (2.9%)	13 (9.6%)	0.340
EFV	2 (5.7%)	12 (8.9%)	0.792
NVP	0	2 (1.5%)	1.000
AZT	0	2 (1.5%)	1.000
ABC	1(2.9%)	0	0.206
Lpv/r	1 (2.9%)	0	0.206
DTG	0	1 (0.7%)	1.000
Elvitegravir	0	1 (0.7%)	1.000
Amphotericin B			
Induction therapy > 0.58 mg/kg/d, n (%)	11 (31.4%)	32 (23.7%)	0.349

*BMI* Body Mass Index, *BUN *Blood urea nitrogen, *AST *Aspartate aminotransferase, *ALT *Alanine aminotransferase, *HAART *Highly active antiretroviral therapy, *3TC *Lamivudine, *TDF *Tenofovir, *EFV *Efavirenz, *NVP *Nevirapine, *AZT *Zidovudine, *ABC *Abacavir, *Lpv/r *Lopinavir and ritonavir, *DTG *Dolutegravir, *FTC* Emtricitabine

**Table 3:** Clinical characteristics of the patients developing severe thrombocytopenia or not at baseline

	Progression to severe thrombocytopenia (*N* = 18)	No progression to severe thrombocytopenia (*N* = 152)	*p*-value
**Socio-demographic**			
Female sex, n (%)	5 (27.8%)	19 (12.5%)	0.161
Age level, median (IQR), year	49 (34–52)	43 (34–52)	0.512
Drink, n (%)	2 (11.1%)	27 (17.8%)	0.705
Smoke, n (%)	4 (22.2%)	44 (28.9%)	0.549
BMI level, median (IQR), kg/m^2^	32 (29–37)	32 (30–35)	0.789
**Symptoms and signs**			
Fever, n (%)	17 (94.4%)	128 (84.2%)	0.419
Cough, n (%)	7 (38.9%)	89 (58.6%)	0.112
Sputum, n (%)	6 (33.3%)	69 (45.4%)	0.330
Hemoptysis, n (%)	0	2 (1.3%)	1.000
Abdominal pain, n (%)	4 (22.2%)	26 (17.1%)	0.832
Diarrhea, n (%)	0	10 (6.6%)	0.554
Headache, n (%)	0	8 (5.3%)	1.000
Skin lesions, n (%)	4 (22.2%)	44 (28.9%)	0.549
Weight loss, n (%)	7 (38.9%)	51 (33.6%)	0.652
**Complications**			
Oral candidiasis, n (%)	5 (27.8%)	67 (44.1%)	0.186
Cytomegalovirus infection, n (%)	1 (5.6%)	16 (10.5%)	0.803
Tuberculosis, n (%)	1 (5.6%)	14 (9.2%)	0.938
Hepatitis B, n (%)	1 (5.6%)	10 (6.6%)	1.000
Pneumocystis pneumonia, n (%)	1 (5.6%)	12 (7.9%)	1.000
Syphilis, n (%)	0	4 (2.6%)	1.000
Hepatitis C, n (%)	1 (5.6%)	0	0.106
Toxoplasma encephalopathy, n (%)	1 (5.6%)	0	0.106
**Laboratory results**			
Leukocyte level, median (IQR), ×10^9^ /L	3.96 (2.67–4.96)	3.96 (2.71–5.53)	0.518
Hemoglobin level, median (IQR), g/L	92 (85–106)	101 (89–112)	0.199
Platelet level, median (IQR), ×10^9^ /L	75 (52–139)	144 (78–217)	0.007
CD4 + T-cell counts, median (IQR), cells/µL	13 (4–23)	15 (7–39)	0.205
Serum creatinine level, median (IQR), µmol/L	65.95 (48.80-78.85)	64.00 (53.93-77.00)	0.871
Total bilirubin level, median (IQR), mg/dl	9.60 (8.38–12.25)	9.28 (6.82–15.98)	0.514
AST/ALT ratio, median (IQR)	1.9 (1.3–4.8)	1.8 (1.3–2.6)	0.254
Sodium level, median (IQR), mmol/liter	134.25 (129.75-136.48)	133.4 (130-137.15)	0.895
Potassium level, median (IQR), mmol/liter	3.58 (3.12–3.88)	3.63 (3.30-4.00)	0.393
**HAART before diagnosis**			
Antiretroviral therapy, n (%)	3 (16.7%)	16 (10.5%)	0.699
3TC	3 (16.7%)	15 (9.9%)	0.630
TDF	3 (16.7%)	11 (7.2%)	0.356
EFV	1 (5.6%)	13 (8.6%)	1.000
NVP	1 (5.6%)	1 (0.7%)	0.201
AZT	0	2 (1.3%)	1.000
ABC	0	1 (0.7%)	1.000
Lpv/r	0	1 (0.7%)	1.000
DTG	1 (5.6%)	0	0.106
Amphotericin B			
Induction therapy > 0.58 mg/kg/d, n (%)	7 (38.9%)	36 (23.7%)	0.264

*BMI* Body Mass Index, *BUN *Blood urea nitrogen, *AST *Aspartate aminotransferase, *ALT *Alanine aminotransferase, *HAART *Highly active antiretroviral therapy, *3TC* Lamivudine, *TDF *Tenofovir, *EFV* efavirenz, *NVP *Nevirapine, *AZT* Zidovudine, *ABC *Abacavir, *Lpv/r* Lopinavir and ritonavir, *DTG *Dolutegravir, *FTC* Emtricitabine

Multivariate logistic regression analysis revealed that hemoglobin levels < 100 g/L (OR = 5.846, 95% CI: 2.765 ~ 12.363), serum creatinine levels > 73.4 µmol/L (OR = 2.573, 95% CI: 1.157 ~ 5.723), AST/ALT ratio > 1.6 (OR = 2.479, 95% CI: 1.167 ~ 5.266), sodium level ≤ 136 mmol/liter (OR = 4.342, 95% CI: 1.747 ~ 10.789), and a dose of amphotericin B deoxycholate > 0.58 mg/kg/d (OR = 2.504, 95% CI:1.066 ~ 5.882) were independent risk factors associated with the development of severe anemia (Table 4).

**Table 4:** Univariate and multivariate analysis of factors associated with severe anemia, severe leukopenia and severe thrombocytopenia for the outcome of HIV-infected patients complicated with talaromyces marneffei infection

Variables			Univariate analysis				Multivariate analysis			
	Tolerance	VIF	β	OR	95% CI	*p*	β	OR	95% CI	*p*
**Progression to severe anemia**										
BMI ≤ 31 kg/m^2^	0.877	1.141	0.776	2.173	1.158 ~ 4.077	0.016				
Weight loss	0.880	1.137	0.975	2.652	1.383 ~ 5.086	0.003				
Hemoglobin level < 100 g/L	0.862	1.161	1.800	6.051	3.081 ~ 11.883	< 0.001	1.766	5.846	2.765 ~ 12.363	< 0.001
Platelet level < 100 × 10^9^ /L	0.815	1.227	0.563	1.756	0.937 ~ 3.292	0.079				
Serum creatinine level > 73.4 µmol/L	0.944	1.059	0.926	2.525	1.290 ~ 4.941	0.007	0.945	2.573	1.157 ~ 5.723	0.021
AST/ALT ratio > 1.6	0.818	1.222	0.985	2.679	1.413 ~ 5.079	0.003	0.908	2.479	1.167 ~ 5.266	0.018
Sodium level ≤ 136 mmol/liter	0.931	1.074	0.663	1.940	1.009 ~ 3.729	0.047	1.468	4.342	1.747 ~ 10.789	0.002
Potassium level ≤ 3.56 mmol/liter, n (%)	0.891	1.123	0.690	1.993	1.070 ~ 3.713	0.030				
Amphotericin B > 0.58 mg/kg/d	0.954	1.048	0.699	2.011	0.999 ~ 4.049	0.050	0.918	2.504	1.066 ~ 5.882	0.035
**Progression to severe leukopenia**										
Tuberculosiss	0.999	1.001	1.064	2.897	0.955 ~ 8.781	0.060	1.196	3.307	1.050 ~ 10.420	0.041
Platelet level (per 10 × 10^9^ /L)	0.999	1.001	-0.049	0.952	0.911 ~ 0.996	0.031	-0.053	0.948	0.905 ~ 0.993	0.024
**Progression to severe thrombocytopenia**										
Male	0.999	1.001	-0.990	0.371	0.119 ~ 1.159	0.088				
Platelet level < 100 × 10^9^ /L	0.999	1.001	1.077	2.935	1.075 ~ 8.016	0.036	1.077	2.935	1.075 ~ 8.016	0.036

*BMI* Body Mass Index, *AST *Aspartate aminotransferase, *ALT *Alanine aminotransferase

Patients co-infected with tuberculosis and those who had lower platelet levels were shown to be at higher risk of progression to severe leukopenia (*p* ≤ 0.1). Co-infection with tuberculosis (OR = 3.307, 95% CI: 1.050 ~ 10.420), and platelet level (per 10 × 109 /L) (OR = 0.952, 95% CI: 0.911 ~ 0.996) were calculated to be independent risk factors associated with the development of severe leukopenia (Table 4).

Male gender and platelet levels lower than 100 × 10^9^ /L were identified as risk factors associated with progression to severe thrombocytopenia (*p* ≤ 0.1). Platelet levels < 100 × 10^9^ /L (OR = 2.935, 95% CI: 1.075 ~ 8.016) was identified as the independent risk factor associated with the development of severe thrombocytopenia (Table 4).

There was no difference in progression to severe anemia, severe leukopenia, and severe thrombocytopenia between the survivors and non-survivors. There was also no difference between the patients without fungal clearance and with fungal clearance at 2 weeks (Table 5).

**Table 5:** Outcomes of the patients developing severe anemia, leukopenia, or thrombocytopenia or not at 2 weeks

	Events		*p*-value	Events		*p*-value
	Survival	Death		Without fungal clearance	Fungal clearance	
Progression to severe anemia						
No, n (%)	96 (57.1)	1 (50.0)	1.000	32 (59.3)	65 (56.0)	0.693
Yes, n (%)	72 (42.9)	1 (50.0)		22 (40.7)	51 (44.0)	
Progression to severe leukopenia						
No, n (%)	133 (79.2)	2 (100.0)	1.000	43 (79.6)	92 (79.3)	0.962
Yes, n (%)	35 (20.8)	0 (0.0)		11 (20.4)	24 (20.7)	
Progression to severe thrombocytopenia						
No, n (%)	150 (89.3)	2 (1000.0)	1.000	45 (83.3)	107 (92.2)	0.079
Yes, n (%)	18 (10.7)	0 (0.0)		9 (16.7)	9 (7.8)	

Eighty-seven patients used that the started dosing for Amphotericin B is 5 mg on the first day, 10 mg on the second day, 20 mg on the third day, and the therapeutic dose is reached on the fourth day. Thirty-five patients used the started dosing for Amphotericin B is 5 mg on the first day, 15 mg on the second day, 25 mg on the third day, and the therapeutic dose is reached on the fourth day. Twenty-seven patients used the started dosing for Amphotericin B is 10 mg on the first day, 20 mg on the second day, and the therapeutic dose is reached on the third day. Comparing with 5 mg on the first day group, 10 mg on the first day group (10 mg, 20 mg, daily) were calculated to be independent risk factors associated with the development of severe anemia (OR = 2.621, 95% CI: 1.107 ~ 6.206) (Table 6).

**Table 6:** Univariate analysis of factors associated with severe anemia, severe leukopenia, and severe thrombocytopenia in 149 patients

Variables	Univariate analysis			
	β	OR	95% CI	*p*
Progression to severe anemia (*n* = 149)				
5 mg on the first day group		1		
10 mg on the first day group	0.963	2.621	1.107 ~ 6.202	0.028
Progression to severe leukopenia (*n* = 149)				
5 mg on the first day group		1		
10 mg on the first day group	0.357	1.429	0.542 ~ 3.769	0.470
Progression to severe thrombocytopenia (*n* = 149)				
5 mg on the first day group				
10 mg on the first day group	0.136	1.146	0.300 ~ 4.376	0.842

5 mg on the first day group: The standard dosing for Amphotericin B is 5 mg on the first day, 10-15 mg on the second day, 20-25 mg on the third day, and the therapeutic dose is reached on the fourth day

10 mg on the first day group: The standard dosing for Amphotericin B is 10 mg on the first day, 20 mg on the second day, and the therapeutic dose is reached on the third day

The group receiving a starting amphotericin B dose (10 mg, 20 mg, daily) exhibited the highest fungal clearance rate at 96.3% (26/27), which was significantly better than the group receiving a starting amphotericin B dose (5 mg, 10 mg, 20 mg, daily) (60.9%) and the group receiving a starting amphotericin B dose (5 mg, 15 mg, 25 mg, daily) (62.9%). No significant differences were observed among the three groups in terms of progression to severe anemia, severe leukopenia, or severe thrombocytopenia at 2 weeks, as well as survival at both 2 weeks and 48 weeks (Table 7).

**Table 7:** Outcomes of three amphotericin B starting regimens at 2 weeks and 48 weeks in 149 patients

Amphotericin B starting regimen	N	Severe anaemia at 2 weeks	Severe leukopenia at 2 weeks	Severe thrombocytopenia at 2 weeks	Fungal clearance at 2 weeks	Survival at 2 weeks	Survival at 48 weeks
5 mg, 10 mg, 20 mg, daily^a^	87	36/87 (41.4%)	16/87 (18.4%)	9/87 (10.3%)	53/87 (60.9%)	85/87 (97.7%)	81/87 (93.1%)
5 mg, 15 mg, 25 mg, daily^a^	25	12/35 (34.3%)	8/35 (22.9%)	3/35 (8.6%)	22/35 (62.9%)	35/35 (100.0%)	33/35 (94.3%)
10 mg, 20 mg, daily^a^	27	17/27 (63.0%)	7/27 (25.9%)	3/27 (11.1%)	26/27 (96.3%)	27/27 (100.0%)	27/27 (100.0%)
*p*	-	0.063	0.661	1.000	0.002	1.000	0.521

^a^Daily amphotericin B dose was the therapeutic dose

**Fig. 2:**
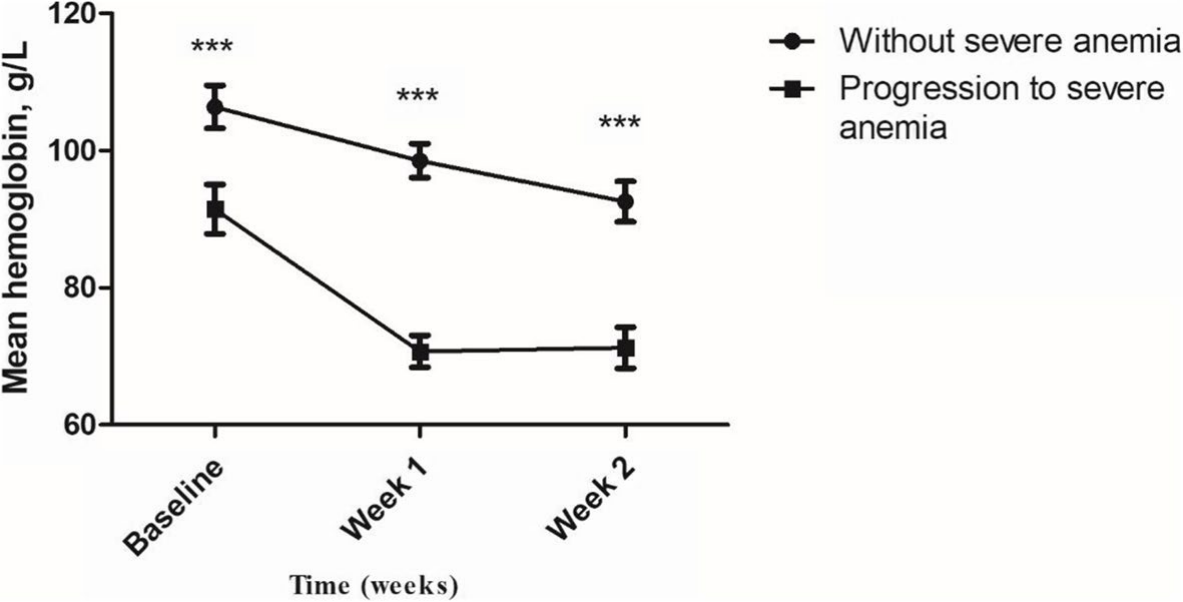
The changes in hemoglobin levels over time were compared between the group without severe anemia and the group with progression to severe anemia. * < 0.05, ** < 0.01, *** < 0.001

Figure 2 shows the changes in hemoglobin levels over time were compared between the group without severe anemia and the group with progression to severe anemia. At baseline, week 1, and week 2, the group that progressed to severe anemia had lower hemoglobin levels compared to the group without severe anemia (median hemoglobin level 91.46 g/L [95% CI, 95.05–87.87 g/L] vs. 106.39 g/L [95% CI, 103.25-109.53 g/L], *p* < 0.001; 70.74 g/L [95% CI, 68.43–73.05 g/L] vs. 98.54 g/L [95% CI, 96.05-101.02 g/L], *p* < 0.001; 71.28 g/L [95% CI, 68.28–74.28 g/L] vs. 92.58 g/L [95% CI, 89.63–95.53 g/L], *p* < 0.001). Eleven patients had hemoglobin levels below 60 g/L within 14 days. Six people changed amphotericin B deoxycholate to other antifungal medications within 14 days, and the median day was day 10 (95% CI: 7.42–12.57 days), but the reason for changing medication was not severe anemia, severe leukopenia, and severe thrombocytopenia. At week 4, the median hemoglobin level of the group that progressed to severe anemia was 87.12 g/L (95% CI, 81.70-92.55 g/L).

## Discussion

Our results observed that the incidence of patients who developed severe anemia, severe leukopenia, and severe thrombocytopenia were 42.9%, 20.6%, and 10.6%, respectively, which approximates the observations of one previous prospective study [3].

A higher AST/ALT level, lower hemoglobin level, higher serum creatinine level, lower sodium level, and a higher administered dose of amphotericin B were found to be independent risk factors for development of severe anemia. AST and ALT are mainly produced in liver cells and are also the main circulating hepatic enzymes in serum. Blood AST and ALT levels could increase as a consequence of hepatocellular damage or hepatic cellular death [3]. Patients with talaromycosis can present with systemic infection, including that of the liver. Thus, hepatic transaminases are often elevated in patients with talaromycosis [16]. One study even observed that a higher AST/ALT ratio increased the risk of death in HIV-infected patients with talaromycosis [1]. However, to our knowledge, no study has investigated the connection between anemia and AST/ALT ratio in HIV-infected patients with talaromycosis. This phenomenon of higher values of AST/ALT associated with a higher prospective risk of severe anemia might be associated with abnormalities in the composition of red blood cell membranes or the limitation of effective bone marrow erythropoiesis, which are known causes of anemia associated with liver disease. Higher serum creatinine levels and lower sodium level are known to be associated with impaired renal function [17]. The preceding study observed that HIV infection and impaired renal function can result in a higher risk of anemia, which concurs with results of our study, as both HIV infection and impaired renal function have a synergistic impact on lowering hemoglobin levels [18]. The adverse effects of Amphotericin B occurs in a dose-dependent manner [13]. This means that higher doses of amphotericin B are associated with a higher risk of adverse effects, which is precisely what we observed in our study. It is, thus, important to establish a balance between maximizing antifungal efficacy and minimizing drug-related toxicity [19].

Patients co-infected with tuberculosis are associated with severe leukopenia. The possible reason for this is that anti-tuberculosis drugs may promote antibody generation and form antigen-antibody complexes which may be absorbed on to leukocytes, and cause leukocyte lysis and damage [20, 21]. Many previous studies have shown that baseline thrombocytopenia is associated with poor prognosis [5, 22]. However, the reason why lower platelet levels at baseline are also associated with severe leukopenia is still unclear.

Lower platelets levels at baseline were found to be associated with the development of severe thrombocytopenia. Talaromycosis itself, together with the toxic effects of amphotericin B deoxycholate, may both promote the development of severe thrombocytopenia. The progression of severe thrombocytopenia is associated with an increased risk of hemorrhage. Discontinuation of the causative drug should sometimes be considered, if necessary.

There was also no difference between the patients without fungal clearance and with fungal clearance at 2 weeks. This means that although decreased baseline hemoglobin, platelets, and white blood cells each are associated with poor prognosis, this does not imply that these severe events are not effectively treated with amphotericin B deoxycholate. The adverse effects of Amphotericin B occur in a dose-dependent manner. Our findings indicate that the group receiving 10 mg on the first day had a higher likelihood of developing severe anemia compared to the group receiving 5 mg on the first day. This suggests that the anemia is likely related to the drug. The majority of patients had completed the 2-week induction therapy based on amphotericin B deoxycholate and stopped amphotericin B deoxycholate at week 2. The group that progressed to severe anemia had a median hemoglobin level of 71.28 g/L [95% CI, 68.28–74.28 g/L] at week 2, which increased to 87.12 g/L (95% CI, 81.70-92.55 g/L) by week 4. This also suggests that the occurrence of anemia may be related to amphotericin B deoxycholate. The erythropoietin suppression by amphotericin B has been proposed to contribute to the development of anemia [23]. we regretted that markers of erythropoietin were not tested.

Starting amphotericin B deoxycholate at a dose of 10 mg on the first day seems to increase the risk of severe anemia but can lead to earlier fungal clearance. If patients are not at risk of developing severe anemia, starting amphotericin B dose (10 mg, 20 mg, daily) is more likely to be beneficial.

This is a subgroup analysis of a prospective multicenter cohort study. Study limitations include missing markers of disseminated intravascular coagulation, hemopoietin, reticulocyte and serum drug concentration determinations, data of some people on the dose escalation of amphotericin B deoxycholate, an exclusively Chinese study cohort, and some seriously ill patients having to be excluded from the study, which limits our study’s overall generalizability.

## Conclusions

The preceding findings reveal risk factors for severe anemia, severe leukopenia, and severe thrombocytopenia. After treatment with Amphotericin B, these severe adverse events are likely unrelated to fungal clearance at 2 weeks. 5 mg on the first day of amphotericin B deoxycholate seems to be able to lower the risk of severe anemia. These findings may contribute to the development of effective prevention and management strategies for patients who are at risk of developing these severe adverse events.

## Data Availability

The datasets used and/or analyzed during the current study are available from the corresponding author on reasonable request.

## References

[1] Qin Y, Zhou Y, Lu Y, Chen H, Jiang Z, He K (2021). Multicentre derivation and validation of a prognostic scoring system for mortality assessment in HIV-infected patients with talaromycosis. Mycoses.

[2] Qin Y, Huang X, Chen H, Liu X, Li Y, Hou J (2020). Burden of Talaromyces marneffei infection in people living with HIV/AIDS in Asia during ART era: a systematic review and meta-analysis. BMC Infect Dis.

[3] Le T, Kinh NV, Cuc NTK, Tung NLN, Lam NT, Thuy PTT (2017). A trial of Itraconazole or Amphotericin B for HIV-Associated Talaromycosis. N Engl J Med.

[4] Zhou Y, Yang Z, Liu M, Lu Y, Qin Y, He X (2020). Independent risk factors for deaths due to AIDS in Chongqing, China: does Age Matter?. Front Med (Lausanne).

[5] Ying RS, Le T, Cai WP, Li YR, Luo CB, Cao Y (2020). Clinical epidemiology and outcome of HIV-associated talaromycosis in Guangdong, China, during 2011–2017. HIV Med.

[6] Ranjana KH, Priyokumar K, Singh TJ, Gupta Ch C, Sharmila L, Singh PN (2002). Disseminated Penicillium marneffei infection among HIV-infected patients in Manipur state, India. J Infect.

[7] Brandriss MW, Wolff SM, Moores R, Stohlman F (1964). Jr. ANEMIA INDUCED BY AMPHOTERICIN B. JAMA.

[8] Belperio PS, Rhew DC (2004). Prevalence and outcomes of anemia in individuals with human immunodeficiency virus: a systematic review of the literature. Am J Med.

[9] Albrecht S, Franzeck FC, Mapesi H, Hatz C, Kalinjuma AV, Glass TR (2019). Age-related comorbidities and mortality in people living with HIV in rural Tanzania. Aids.

[10] Haider BA, Spiegelman D, Hertzmark E, Sando D, Duggan C, Makubi A (2019). Anemia, Iron Deficiency, and Iron Supplementation in Relation to Mortality among HIV-Infected patients receiving highly active antiretroviral therapy in Tanzania. Am J Trop Med Hyg.

[11] Yeaman MR (1997). The role of platelets in antimicrobial host defense. Clin Infect Dis.

[12] Madzime M, Rossouw TM, Theron AJ, Anderson R, Steel HC (2021). Interactions of HIV and antiretroviral therapy with neutrophils and platelets. Front Immunol.

[13] Lemke A, Kiderlen AF, Kayser O, Amphotericin B (2005). Appl Microbiol Biotechnol.

[14] Falci DR, da Rosa FB, Pasqualotto AC (2015). Hematological toxicities associated with amphotericin B formulations. Leuk Lymphoma.

[15] Zhou Y, Qin Y, Lu Y (2022). Efficacy and safety of Voriconazole Versus amphotericin B deoxycholate induction treatment for HIV-Associated Talaromycosis: a prospective Multicenter Cohort Study in China. Infect Dis Ther.

[16] Le T, Wolbers M, Chi NH, Quang VM, Chinh NT, Lan NP (2011). Epidemiology, seasonality, and predictors of outcome of AIDS-associated Penicillium marneffei infection in Ho Chi Minh City, Viet Nam. Clin Infect Dis.

[17] Kannapiran M, Nisha D, Madhusudhana Rao A (2010). Underestimation of impaired kidney function with serum creatinine. Indian J Clin Biochem.

[18] Abraham AG, Palella FJ, Li X, Estrella MM, Kingsley LA, Witt MD (2012). The impact of impaired kidney function and HIV infection on the risk of anemia. AIDS Res Hum Retroviruses.

[19] Le T, Ly VT, Thu NTM, Nguyen A, Thanh NT, Chau NVV, et al. Population Pharmacodynamics of Amphotericin B Deoxycholate for disseminated infection caused by Talaromyces marneffei. Antimicrob Agents Chemother. 2019;63(2):e01739-18. 10.1128/AAC.01739-18 PMC6355582 30420478

[20] De Vriese AS, Robbrecht DL, Vanholder RC, Vogelaers DP, Lameire NH (1998). Rifampicin-associated acute renal failure: pathophysiologic, immunologic, and clinical features. Am J Kidney Dis.

[21] Lin FS, Wu MY, Tu WJ, Pan HQ, Zheng J, Shi JW (2015). A cross-sectional and follow-up study of leukopenia in tuberculosis patients: prevalence, risk factors and impact of anti-tuberculosis treatment. J Thorac Dis.

[22] Shi M, Lin J, Wei W (2022). Machine learning-based in-hospital mortality prediction of HIV/AIDS patients with Talaromyces marneffei infection in Guangxi, China. PLoS Negl Trop Dis.

[23] Yeo EJ, Ryu JH, Cho YS (2006). Amphotericin B blunts erythropoietin response to hypoxia by reinforcing FIH-mediated repression of HIF-1. Blood.

